# Strategies of *Nitrosomonas europaea *19718 to counter low dissolved oxygen and high nitrite concentrations

**DOI:** 10.1186/1471-2180-10-70

**Published:** 2010-03-04

**Authors:** Ran Yu, Kartik Chandran

**Affiliations:** 1 Department of Earth and Environmental Engineering, Columbia University, New York, NY 10027, USA

## Abstract

**Background:**

*Nitrosomonas europaea *is a widely studied chemolithoautotrophic ammonia oxidizing bacterium. While significant work exists on the ammonia oxidation pathway of *N. europaea*, its responses to factors such as dissolved oxygen limitation or sufficiency or exposure to high nitrite concentrations, particularly at the functional gene transcription level are relatively sparse. The principal goal of this study was to investigate responses at the whole-cell activity and gene transcript levels in *N. europaea *19718 batch cultures, which were cultivated at different dissolved oxygen and nitrite concentrations. Transcription of genes coding for principal metabolic pathways including ammonia oxidation (*amoA*), hydroxylamine oxidation (*hao*), nitrite reduction (*nirK*) and nitric oxide reduction (*norB*) were quantitatively measured during batch growth, at a range of DO concentrations (0.5, 1.5 and 3.0 mg O_2_/L). Measurements were also conducted during growth at 1.5 mg O_2_/L in the presence of 280 mg-N/L of externally added nitrite.

**Results:**

Several wide ranging responses to DO limitation and nitrite toxicity were observed in *N. europaea *batch cultures. In contrast to our initial hypothesis, exponential phase mRNA concentrations of both *amoA *and *hao *increased with decreasing DO concentrations, suggesting a mechanism to metabolize ammonia and hydroxylamine more effectively under DO limitation. Batch growth in the presence of 280 mg nitrite-N/L resulted in elevated exponential phase *nirK *and *norB *mRNA concentrations, potentially to promote utilization of nitrite as an electron acceptor and to detoxify nitrite. This response was in keeping with our initial hypothesis and congruent with similar responses in heterotrophic denitrifying bacteria. Stationary phase responses were distinct from exponential phase responses in most cases, suggesting a strong impact of ammonia availability and metabolism on responses to DO limitation and nitrite toxicity. In general, whole-cell responses to DO limitation or nitrite toxicity, such as sOUR or nitrite reduction to nitric oxide (NO) did not parallel the corresponding mRNA (*nirK*) profiles, suggesting differences between the gene transcription and enzyme translation or activity levels.

**Conclusions:**

The results of this study show that *N. europaea *possesses specific mechanisms to cope with growth under low DO concentrations and high nitrite concentrations. These mechanisms are additionally influenced by the physiological growth state of *N. europaea *cultures and are possibly geared to enable more efficient substrate utilization or nitrite detoxification.

## Background

*Nitrosomonas europaea *is a widely studied chemolithoautotrophic ammonia oxidizing bacterium (AOB) that catalyzes the aerobic oxidation of ammonia (NH_3_) to nitrite (NO_2_^-^) using carbon dioxide (CO_2_) as the preferred assimilative carbon source [1]. Bacteria closely related to *N. europaea *have been found in various natural and engineered environments indicating that they can proliferate under different growth conditions, by effectively utilizing growth substrates such as NH_3 _and oxygen [2-4].

**Figure 1:**
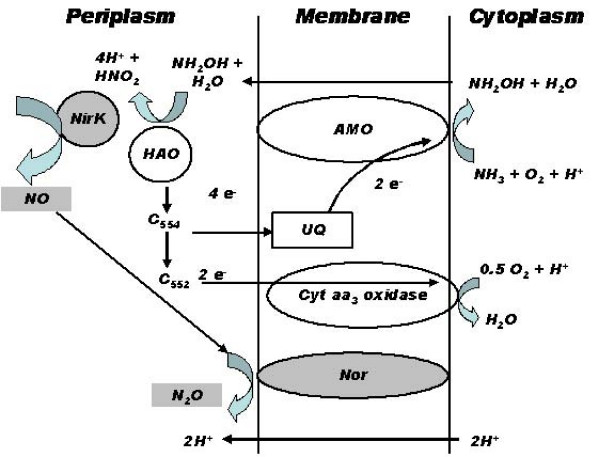
**Schematic of oxidative (unshaded enzymes) and reductive (gray shaded enzymes) nitrogen transformations in *N. europaea *(modified after **[5]**)**.

The oxidative catabolic pathway of *N. europaea *involves NH_3 _oxidation to hydroxylamine (NH_2_OH) by membrane bound ammonia monooxygenase (AMO) and NH_2_OH oxidation to NO_2_^- ^by periplasmic hydroxylamine oxidoreductase (HAO) (Figure 1) [5]. In addition, autotrophic denitrification by *N. europaea *has also been shown [6-8]. It is believed that denitrification by *N. europaea *is especially favored during growth under low dissolved oxygen (DO) concentrations or high nitrite concentrations [9] and results in the production of nitric oxide (NO) or nitrous oxide (N_2_O) [10,11]. However, little information exists on the mechanisms driving the responses of *N. europaea *to DO limitation and possible NO_2_^- ^toxicity [12]. For instance, it is as yet unknown whether responses to DO limitation and NO_2_^- ^toxicity at the whole-cell level are ultimate manifestations of changes in gene transcription and expression.

In this study, the ability of *N. europaea *to transcribe four key genes involved in its catabolic pathway as a function of batch growth conditions (NH_3 _sufficiency and starvation, DO limitation and NO_2_^- ^toxicity) was evaluated. It was hypothesized that DO limitation and NO_2_^- ^toxicity would result in lower transcription of genes coding for NH_3 _and NH_2_OH oxidation (*amoA *and *hao*, respectively), given that these are the main steps leading to energy generation in *N. europaea *[5]. Furthermore, given that low DO and high NO_2_^- ^concentrations are two main triggers for expression of denitrification genes in heterotrophic bacteria [13], it was hypothesized that decreasing DO concentrations and high NO_2_^- ^concentrations would similarly induce progressively higher transcription of NO_2_^- ^and NO reductase genes in *N. europaea *(*nirK *and *norB*, respectively).

The specific objectives of this study were to (i) quantitatively measure the transcription of *amoA, hao, nirK *and *norB*, four genes involved in redox N transformations, in *N. europaea *during batch growth at different DO and initial NO_2_^- ^concentrations; and (ii) compare gene transcription level responses to DO limitation and NO_2_^- ^inhibition with 'whole-cell' responses related to activity, biokinetics and production of gaseous NO- the first product of sequential NO_2_^- ^reduction by *N. europaea*.

## Results

### Impact of reactor DO on N speciation, biokinetics and functional gene transcription

**Figure 2:**
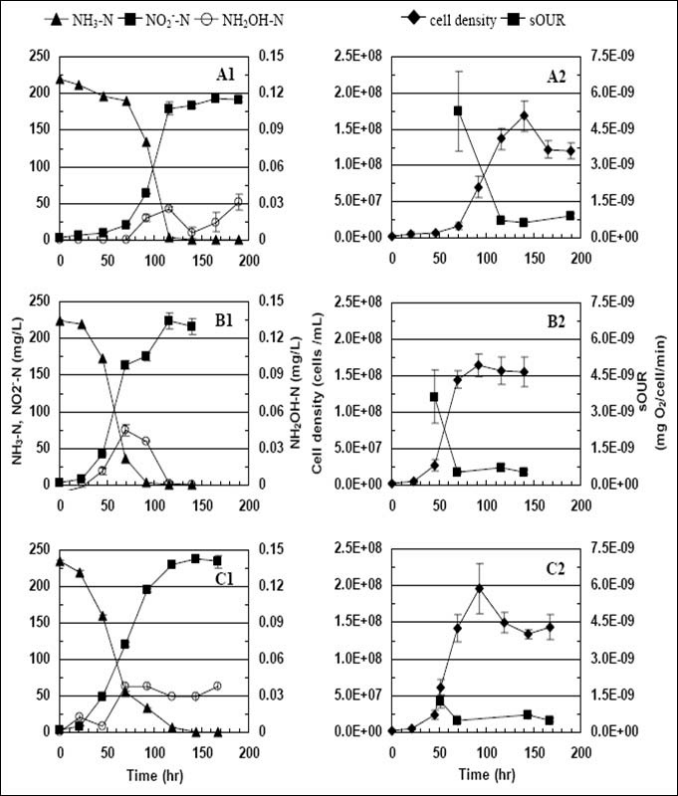
**NH_3_-N, NO_2_^-^-N, and NH_2_OH-N, (A1-C1), cell density and sOUR (A2-C2) profiles during *N. europaea *batch growth at DO = 0.5 mg/L (A), 1.5 mg/L (B) and 3 mg/L (C)**.

Batch cultivation of *N. europaea *cultures at different DO concentrations (0.5, 1.5 and 3.0 mg O_2_/L) led to several differences at the nitrogen speciation, biokinetics and gene transcription levels. Based on a studentized t-test, the degree of NH_3_-N conversion to NO_2_^-^-N at DO = 0.5 mg O_2_/L (76 ± 16%) was significantly lower (p < 0.05) than at DO = 1.5 mg O_2_/L, (90 ± 10%) or DO = 3.0 mg O_2_/L (89 ± 15%), respectively, (Figure 2, A1-C1). The final cell concentrations were relatively uniform for all three DO concentrations (Figure 2, A2-C2). However, the lag phase at DO = 0.5 mg O_2_/L was one day longer than at DO = 1.5 or 3.0 mg O_2_/L pointing to the impact of electron acceptor limitation on the cell synthesizing machinery of *N. europaea *(Figure 2, A2-C2). Estimates of the maximum specific growth rate (obtained via non-linear estimation [14]) at DO = 0.5 mg O_2_/L (0.043 ± 0.005 h^-1^), 1.5 mg O_2_/L (0.057 ± 0.012 h^-1^) and 3.0 mg O_2_/L (0.060 ± 0.011 h^-1^) were not statistically different at α = 0.05. At all three DO concentrations tested, low levels of NH_2_OH transiently accumulated in the growth medium during the exponential phase, in keeping with its role as an obligate intermediate of NH_3 _oxidation [5] (Figure 2, A1-C1). The initial increase in NH_2_OH concentrations at DO = 0.5 mg O_2_/L, was the slowest, due to the longer lag-phase (Figure 2, A1). The peak NH_2_OH concentration at DO = 0.5 mg O_2_/L was also lower than at DO = 1.5 or 3.0 mg O_2_/L (Figure 2, A1-C1).

The peak 'potential' biokinetics of NH_3 _oxidation (expressed as sOUR, and measured under non-limiting DO and ammonia concentrations) varied inversely with reactor DO concentrations (Figure 2, A2-C2). sOUR values consistently peaked during early exponential growth phase followed by a significant decrease during stationary phase (Figure 2, A2-C2), in good correspondence with recent results [15]. Additional sOUR assays could not be conducted during the lag phase, owing to low cell concentrations, which would have consequently necessitated removal of excessively high sampling volumes.

**Figure 3:**
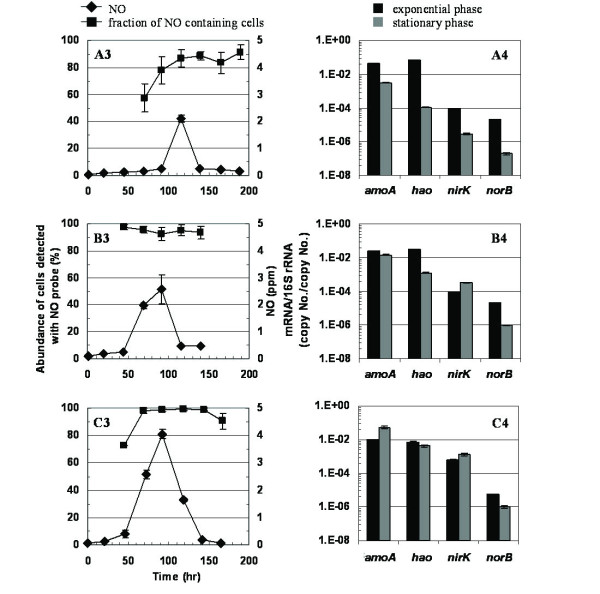
**NO profiles and fraction of NO containing cells (A3-C3), and gene expression (A4-C4) during exponential phase and stationary phase at DO = 0.5 mg/L (A), 1.5 mg/L (B) and 3 mg/L (C) for cultures shown in Figure 2**.

Headspace NO concentrations peaked during the exponential phase and significantly diminished upon NH_3 _exhaustion in the stationary phase (Figure 3, A3-C3). An increasing trend in peak headspace NO concentrations was observed with increasing DO concentrations. NO formation was strictly biological and was not observed in cell-free controls (data not shown). At all DO concentrations tested, intracellular NO was detected in the majority of sampled cells (Figure 3 A3-C3). NO 'positive' cell concentrations were highest especially during late exponential and stationary phases when NO_2_^-^, the likely substrate for NO production, concentrations were the highest (Figure 3 A3-C3). The more gradual increase in the proportion of NO positive cells at DO = 0.5 mgO_2_/L paralleled the trend in peak headspace NO concentrations (Figures 2, 3).

The impact of operating DO concentrations on gene transcript profiles, determined using primer sets described in Table 1, was dependent upon the physiological growth phase. In exponential phase cell samples, *amoA *and *hao *relative mRNA concentrations *statistically decreased *with *increasing *reactor DO concentrations (Figure 3, A4-C4, Table 2). A systematic impact of growth phase on *nirK *and *norB *relative mRNA concentrations was not observed during exponential phase. The relative mRNA concentrations for both genes during exponential phase were statistically similar for DO = 0.5 and 1.5 mg O_2_/L and statistically higher (for *nirK*) or lower (for *norB*) at DO = 3.0 mg O_2_/L (Figure 3, A4-C4, Table 2). In direct contrast, during stationary phase, the relative mRNA concentrations of *amoA, hao *and *nirK *all *statistically increased *with *increasing *DO concentrations. Additionally, the relative mRNA concentrations of *norB *at DO = 1.5 mg O_2_/L were statistically higher than at DO = 0.5 mg O_2_/L, but statistically similar to those at DO = 3.0 mg O_2_/L (Table 2).

**Table 1:** Endpoint and real-time PCR primers employed in this study

Primer	Sequence (5'-3')	Position	Target gene	Reference
Endpoint PCR				
A189 amoA2R'	GGHGACTGGGAYTTCTGG CCTCKGSAAAGCCTTCTTC	151-168 802-820	*amoA*	[36,37]
HAO1F HAO1R	TCAACATAGGCACGGTTCATCGGA ATTTGCCGAACGTGAATCGGAACG	203-226 1082-1105	*hao*	[38]
NirK1F NirK1R	TGCTTCCGGATCAGCGTCATTAGT AGTTGAAACCGATGTGGCCTACGA	31-54 809-832	*nirK*	[38]
NorB1F NorB1R	CGGCACTGATGTTCCTGTTTGCTT AGCAACCGCATCCAGTAGAACAGA	479-502 1215-1238	*norB*	[38]
KNO50F KNO51R	TNANACATGCAAGTCGAICG GGYTACCTTGTTACGACTT	49-68 1492-1510	Eubacterial 16S rRNA gene	[39]

Quantitative PCR				
amoAFq amoARq	GGACTTCACGCTGTATCTG GTGCCTTCTACAACGATTGG	408-426 524-543	*amoA*	[15]
HAO1Fq HAO1Rq	TGAGCCAGTCCAACGTGCAT AAGGCAACAACCCTGCCTCA	266-285 331-350	*hao*	[38]
NirK1Fq NirK1Rq	TGCAGGGCATACTGGACGTT AGGTGAACGGGTGCGCATTT	182-201 291-310	*nirK*	[38]
NorB1Fq NorB1Rq	ACACAAATCACTGCCGCCCA TGCAGTACACCGGCAAAGGT	958-977 1138-1157	*norB*	[38]
EUBF EUBR	TCCTACGGGAGGCAGCAGT GGACTACCAGGGTATCTAATCCTGTT	339-357 780-805	Eubacterial 16S rRNA gene	[34]

**Table 2:** Statistical comparison of the impact of DO concentrations on relative mRNA concentrations in exponential (E) and stationary (S) phase cultures (p values < 5.0 × 10^-2 ^indicate statistically significant differences).

DO (mg O_2_/L) comparison	p =							
	
	*amoA*		*hao*		*nirK*		*norB*	
	E	S	E	S	E	S	E	S
**0.5 -- 1.5**	1.32 × 10^-4^	**1.64 × 10^-5^**	4.86 × 10^-5^	**3.3 × 10^-5^**	9.48 × 10^-1^	**2.9 × 10^-5^**	6.29 × 10^-1^	**4.63 × 10^-6^**

**1.5 -- 3.0**	1.2 × 10^-5^	**1.98 × 10^-3^**	2.26 × 10^-11^	**2.16 × 10^-3^**	1.22 × 10^-5^	**1.78 × 10^-3^**	1.83 × 10^-7^	7.52 × 10^-1^

Underlined text indicates statistically similar results, bold text indicates statistical increase and regular text indicates decrease.

At DO = 0.5 mg O_2_/L, the transition from exponential phase to stationary phase resulted in a systematic decrease in relative mRNA concentrations of all four genes (Figure 3A4-C4 and Table 3). At DO = 1.5 mg O_2_/L, this trend was valid for *amoA, hao *and *norB*. However, the stationary phase *nirK *relative mRNA concentrations were statistically higher than during exponential phase. At DO = 3.0 mg O_2_/L, only *hao *and *norB *displayed a decrease in relative mRNA concentrations upon transition from exponential to stationary phase (Figure 3A4-C4, Table 3). In contrast, relative mRNA concentrations of *amoA *and *nirK *increased during stationary phase (Figure 3A4-C4, Table 3). Additionally, except at DO = 1.5 mg O_2_/L for *nirK*, the relative retention of *amoA *mRNA concentrations during stationary phase relative to exponential phase was consistently the highest (Figure 3 B4-C4). The retention factors averaged across all three DO concentrations were 1.98:1, 0.21:1, 1.86:1 and 0.08:1 for *amoA*, *hao*, *nirK *and *norB*, respectively (where a retention factor > 1) suggests relative increase during stationary phase).

**Table 3:** Statistical comparison of relative mRNA concentrations and sOUR in exponential and stationary phase cultures grown at different DO concentrations (p values < 5.0 × 10^-2 ^indicate statistically significant differences).

DO (mg O_2_/L)	p =				
	
	*amoA*	*hao*	*nirK*	*norB*	sOUR
**0.5**	5.0 × 10^-5^	1.1 × 10^-5^	3.2 × 10^-6^	8.0 × 10^-6^	5.0 × 10^-1^

**1.5**	5.5 × 10^-6^	6.4 × 10^-8^	**7.7 × 10^-5^**	3.9 × 10^-6^	1.5 × 10^-3^

**3.0**	**1.5 × 10^-3^**	6.3 × 10^-4^	**5.1 × 10^-3^**	1.0 × 10^-6^	1.2 × 10^-1^

Underlined text indicates statistically similar results, bold text indicates statistical increase and regular text indicates decrease.

### Impact of growth in the presence of added nitrite on N speciation, biokinetics and gene transcription

**Figure 4:**
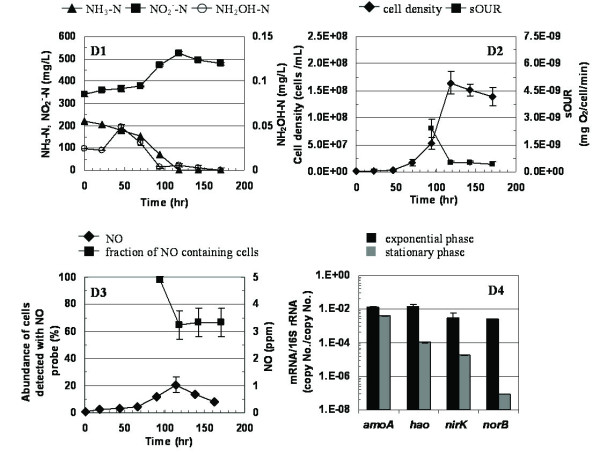
**Profiles of NH_3_-N, NO_2_^-^-N, and NH_2_OH-N (D1), cell density and sOUR (D2), NO and fraction of NO containing cells (D3) and gene expression (D4) during exponential phase and stationary phase at DO = 1.5 mg/L in the presence of added 280 mg NO_2_^-^-N/L**.

Cell growth was not detected at an initial NO_2_^- ^concentration of 560 mg-N/L and DO = 1.5 mg O_2_/L, even after 2 weeks of incubation (data not shown). An initial NO_2_^- ^concentration of 280 mg NO_2_^-^-N/L and DO = 1.5 mg O_2_/L, resulted in a lag phase one day longer than that in the initial absence of nitrite (Figure 4 D1-D2 and Figure 2, B1-B2, respectively). However, the overall cell yield was not impacted. The extent of NH_3 _oxidized to NO_2_^- ^in the presence of 280 mg NO_2_^-^-N/L (88 ± 5%, n = 2) was not significantly different (α = 0.05) than in the absence of nitrite (90 ± 10%, n = 2). NH_2_OH accumulation was observed during the extended lag phase suggesting initial inhibition of NH_2_OH oxidation by NO_2_^- ^(Figure 4, D1). Lower NO production was observed in the presence of added NO_2_^- ^(Figure 4, D3). In parallel, a substantial reduction in the fraction of cells with detectable intracellular NO was also observed, from 98.3 ± 2.1% during exponential phase to 66.6 ± 10.4% during stationary phase (Figure 4, D3). sOUR values were not significantly different (α = 0.05) in the presence or absence of added NO_2_^-^-N/L (Figure 4, D2, Figure 2, B2, respectively). Exponential phase relative mRNA concentrations of *amoA *and *hao *were statistically lower during growth in the presence of 280 mg NO_2_^-^-N/L than in the absence of added nitrite (Figure 4, D4, Table 4). However, exponential phase transcription of *nirK *and *norB *was significantly higher in the presence of 280 mg NO_2_^-^-N/L than in the absence of added nitrite (Figure 4, D4 and Figure 3, B4, Table 4). During stationary phase, *amoA*, *hao, nirK *and *norB *relative mRNA concentrations were all statistically lower in the presence of 280 mg NO_2_^-^-N/L than in the absence of added nitrite (Figure 3, B4 and Figure 4, D4, Table 4).

**Table 4:** Statistical comparison of relative mRNA concentrations and sOUR in exponential (E) and stationary (S) phase cultures grown in the presence and absence of nitrite (p values < 5.0 × 10^-2 ^indicate statistically significant differences).

Growth phase	p =				
	
	*amoA*	*hao*	*nirK*	*norB*	sOUR
**E**	7.9 × 10^-4^	1.2 × 10^-3^	**1.3 × 10^-3^**	**2.8 × 10^-3^**	7.0 × 10^-3^

**S**	5.1 × 10^-5^	3.2 × 10^-5^	3.2 × 10^-5^	4.6 × 10^-5^	2.0 × 10^-1^

Underlined text indicates statistically similar results, bold text indicates statistical increase and regular text indicates decrease.

## Discussion

### Functional gene transcription and N profiles during batch growth of *N. europaea*

In addition to its well-studied NH_3 _oxidation pathway, the genome of *N. europaea *contains genes coding for several denitrification steps, including NO_2_^- ^and NO reduction [16]. While significant work exists on expression analysis of *amoA *and to an extent, *hao*, [17-22], quantitative transcription patterns for *nirK *and *norB *are relatively less characterized. The significance of this study therefore lies in elucidating the co-transcription patterns of *amoA, hao*, *nirK *and *norB *under varying degree of DO and NO_2_^- ^exposure during batch growth of *N. europaea*.

The general overall reduction in *amoA *transcription during the stationary phase, at DO = 0.5 and 1,5 mg O_2_/L (Figure 3, A4-B4), can be linked to dwindling energy resources for *N. europaea *[15,23] or toxicity of accumulating NO_2_^- ^concentrations [21]. The higher *amoA *relative mRNA concentrations during the stationary phase at DO = 3.0 mg O_2_/L were not expected and likely due to the opposing trends in exponential phase and stationary phase responses to increasing DO concentrations (Figure 3, B4-D4), as discussed below.

The retention of relatively higher *amoA *mRNA concentrations during stationary phase compared to those for *hao*, *nirK *and *norB *points to the capability of *N. europaea *to sustain and rapidly increase NH_3 _oxidation during a transition from a starvation state (as in stationary phase) to when NH_3 _becomes available. Since NH_3 _oxidation is the very first step in energy generation for *N. europaea*, it is indeed advantageous to retain the capability (by retaining *amoA *mRNA) for this step to a certain extent compared to downstream steps. These results are consistent with the higher retention of *amoA *mRNA concentrations relative to those for other genes coding for carbon dioxide fixation for growth, ion transport, electron transfer and DNA replication [23]. In fact, an actual *increase *in NH_3 _transport genes during NH_3 _starvation in stationary phase has also been observed [23].

The increasing trend in relative mRNA concentrations of *amoA *and *hao *and sOUR with decreasing DO concentrations during exponential growth reflect a possible strategy of *N. europaea *to (partially) make up for low DO concentrations by enhancing the ammonia and hydroxylamine oxidizing machinery. One possible means to enhance substrate utilization rates at reduced DO concentrations could be to increase the capacity for oxygen transfer into the cell itself.

An alternate means could be by enhancing the ammonia or hydroxylamine oxidizing machinery (mRNA, proteins and or protein activity). The volumetric ammonia oxidation rate depends upon the mathematical product of AMO (or HAO) protein concentrations, their activity and DO concentrations (as given by the multiplicative Monod model [24]). Therefore, potentially similar ammonia oxidation rates could be maintained at lower DO concentrations by increasing the catalytic protein concentrations (or those of their precursors, such as mRNA) or activities (as measured by sOUR assays). Such an enhancement might be manifested in higher 'potential' oxygen uptake rates, measured under non-limiting DO concentrations. Notwithstanding increased 'potential' NH_3 _or NH_2_OH oxidation activity from cells exposed to sustained lower DO concentrations, actual 'extant' activity is indeed expected to be lower under stoichiometric DO limitation, resulting in lower rates of batch cell growth or nitrite accumulation (Figure 2, A2-C2). Based on a recent study, *N. europaea *cultures demonstrated similar increases in *amoA *transcription and sOUR when subject to NH_3 _limitation in chemostats, relative to substrate sufficient batch cultures [15].

While it is documented that NirK is involved in NH_3 _oxidation by facilitating intermediate electron transport [25], the specific role of the Nor cluster in NH_3 _metabolism and exclusivity in N_2_O prodution is unclear [7]. Both NirK and Nor act upon products of upstream AMO and HAO. Thus, the lack of systematic trends in relative mRNA concentrations of *nirK *or *norB *with changing DO concentrations possibly point to less stringent regulation of these two genes during exponential growth in the overall catabolic pathways of *N. europaea*.

In contrast to exponential phase, the statistical increase in relative mRNA concentrations with increasing DO concentrations for all four genes during stationary phase is clearly intriguing. These trends highlight the impact of starvation on responses to different DO concentrations. Although the unique responses of *N. europaea *to starvation [23] and oxygen concentrations (via Fnr [26]) have been documented, the mechanisms of combined NH_3 _and DO based gene regulation in *N. europaea *are not well understood. It is well documented that ammonia oxidizing bacteria, such as *N. europaea*, are commonly subject to cycling between anoxic and oxic conditions and a wide range of NH_3 _concentrations in engineered and natural environments such as wastewater treatment plants or soils [24,27,28]. The specific responses observed herein might be part of a coordinated strategy of *N. europaea *to maintain active or latent substrate metabolic machinery to counter such varying environments and clearly merit further study.

The differences in observed transient accumulation of NH_2_OH could also be explained at the transcription and protein activity levels. The decrease in exponential phase *hao *relative mRNA concentrations with increasing DO was more rapid than for *amoA *(Figure 3 A4-C4). This decrease coupled with a decrease in sOUR (a composite measure of AMO and HAO activity) with increasing DO, could have resulted in the observed trends in NH_2_OH concentrations. Although it has been shown that *N. europaea *can retain high levels of HAO protein and activity under ammonia starvation [29], the impact of DO concentrations on HAO activity has not been specifically identified. While the gene transcript data provide good insights into possible responses of *N. europaea *to different DO concentrations, protein activity data is crucial to explain profiles of intermediates such as NH_2_OH.

The parallel profiles of exponential phase *nirK *relative mRNA concentrations and headspace NO concentrations at different DO concentrations (Figure 3) suggest a possible link between *nirK *transcription and NO generation. However, the loss of this parallel in the presence of added NO_2_^- ^(higher *nirK *gene transcription but lower NO concentrations, Figure 4) suggests the possible presence of NO generation pathways that are distinct from NO_2_^- ^reduction, as pointed out previously [26] or even post-transcriptional effects. Indeed, there is still no consensus about the source of NO produced by AOB, such as *N. europaea*, and the potential roles of *nirK*, *hao *and a multicopper oxidase of the *nirK *operon have all been implicated [26].

### Impact of exposure to high nitrite concentrations on gene transcription

High NO_2_^- ^concentrations have been implicated as the principal trigger for high NirK protein activity in *N. europaea *[9], which has a fundamental grounding in the similar trends observed in this study at the *nirK *gene mRNA level during exponential growth (Figure 4 D4). Increased *nirK *transcription is the result of the regulatory activity of the NsrR repressor protein, which is present in the genome of *N. europaea *[16]. NsrR is responsible for sensing NO and NO_2_^- ^concentrations and is supposedly involved in the transcriptional regulation of several operons including the *nirK *gene cluster of *N. europaea *[9]. Although *N. europaea *contains *norB*, alternate pathways are possibly involved in the production of N_2_O [7], the increased transcription of *norB*, shown in this study cannot be unequivocally reconciled with functional N_2_O production. Nevertheless, the increased transcription of both *nirK *and *norB *in response to high nitrite concentrations is in keeping with one of our initial hypotheses.

The uniformly lower transcript concentrations upon growth with added 280 mg NO_2_^-^-N/L could be a result of energy resources channeled towards mitigation of nitrite toxicity rather than its utilization as an electron acceptor during stationary phase. In general, it could be argued that in response to nitrite toxicity during ammonia starvation, there is little incentive to increase transcription of putative nitrite and nitric oxide reduction pathways. However, it should be noted that the lower transcript abundance during stationary phase when grown with added 280 mg NO_2_^-^-N/L is in direct contrast to an increase in *nirK *during stationary phase, when grown without added NO_2_^-^-N (Figure 3 B4-C4). The more gradual build-up of nitrite in the latter case could have allowed for adaptation, whereas the initial spike of 280 mg NO_2_^-^-N/L might have imposed a significant toxic stress that resulted in reduced growth and different transcriptional profiles. Indeed, the toxic stress was possibly too severe at 560 mg NO_2_^-^-N/L, which resulted in no growth whatsoever.

Additionally, the reduction in transcript abundance of *amoA *and *hao *in the presence of NO_2_^-^-N, did not parallel the relatively unchanged sOUR in the presence or absence of NO_2_^-^-N. Given that sOUR is a measure of the sum of AMO and HAO activities, these results also suggest uncoupling of the responses at the gene transcription and post-transcriptional or translational levels (Figure 4). Responses at the protein abundance and activity levels would be needed to substantiate and provide an explanation for such uncoupling.

It should be noted that the severe impacts of added nitrite were possibly related to the application of these high nitrite concentrations at the beginning of the batch growth assays. Had the nitrite concentrations been applied during periods of relatively higher cell concentrations (during exponential or stationary phase), the impacts might have been less severe, given that the cells were already producing and responding to the increasing NO_2_^-^-N levels in the culture medium. Thus, in a sense, the results reported herein represent the most extreme response of *N. europaea *cultures to nitrite exposure.

## Conclusions

The responses of *N. europaea *to cope with DO limitation and NO_2_^- ^toxicity were wide-ranging from the gene transcription through whole cell levels. The results refuted the initial hypothesis that low DO is one of the main pre-requisite conditions for the transcription of *nirK *and *norB *genes in *N. europaea*. On the other hand, these results indeed supported our other hypothesis that higher NO_2_^- ^concentrations constitute the principal trigger for increased relative transcription related to autotrophic denitrification reactions. The distinct responses observed during the exponential and stationary phase to both DO limitation and nitrite toxicity highlight the need to understand the specific regulatory mechanisms employed by *N. europaea *to jointly counter substrate starvation and stress.

## Methods

### Cultivation of batch *N. europaea *cultures

*N. europaea *(ATCC 19718, Manassas, VA) batch cultures were cultivated in the dark in batch bioreactors (Bellco Glass, Vineland, NJ, working volume = 4 L, agitation speed = 200 rpm) in a growth medium containing 280 mg-N/L and in addition (per liter): 0.2 g of MgSO47H_2_O, 0.02 g of CaCl_2_2H_2_O, 0.087 g of K_2_HPO_4_, 2.52 g EPPS (3- [4-(2-Hydroxyethyl)-1-piperazine] propanesulfonic acid), 1 mL of 13% EDTA-Fe^3+^, 1 mL of trace elements solution (10 mg of Na_2_MoO_4_2H_2_O, 172 mg of MnCl_2_4H_2_O, 10 mg of ZnSO_4_7H_2_O, 0.4 mg of CoCl_2_6H_2_O, and 100 mL of distilled water), 0.5 mL of 0.5% phenol red, and 0.5 mL of 2 mM CuSO_4_5H_2_O. Reactor pH was controlled in the range 6.8-7.4 by manual addition of pre-sterilized 40% potassium bicarbonate solution.

Batch growth experiments were conducted at three DO concentrations, 0.5 ± 0.05, 1.5 ± 0.05 and 3.0 ± 0.05 mg O_2_/L. Batch reactor DO was measured and controlled with a fermentation DO probe and benchtop dissolved oxygen meter and controller system (Cole-Parmer, Vernon Hills, IL) using a combination of filter sterilized (0.2 μm pore size, Millipore^®^, Ann Arbor, MI) nitrogen gas or air. In select experiments conducted at DO = 1.5 ± 0.05 mg O_2_/L, the feed medium additionally contained 280, or 560 mg NO_2_^-^-N/L before *N. europaea *inoculation, which enabled the determination of batch growth in the presence of these high NO_2_^-^-N concentrations. NH_3 _(gas-sensing electrode, Corning, Corning, NY), NH_2_OH [30], NO_2_^- ^(diazotization, [31], cell concentration (direct counting) and gaseous NO (chemiluminescence, CLD-64, Ecophysics, Ann Arbor, MI) were measured once a day during the batch growth profile. All batch growth experiments were conducted in duplicate.

### Detection of intracellular and extracellular nitric oxide

Intracellular NO presence was determined by staining with 4-amino-5-methylamino-2',7'-difluorofluorescein diacetate (Molecular Probes, Eugene, OR) for 30 min in the absence of light. Stained cells were washed twice with sterile NH_3_-free medium and quantified immediately with epifluorescence microscopy (Nikon ECLIPSE 80 *i*) using a minimum of 10 randomly-chosen microscopic fields (each 0.30 × 0.22 mm^2^). NO was specifically the focus of gaseous bulk phase and intra-cellular measurements since it is the direct product of nitrite reduction, the main focus of this study. Additionally, the presence of NO inside *N. europaea *cells strongly implicates its direct production by the cells themselves rather than by extracellular abiotic reactions. In contrast to NO, there is currently no method that allows detection of intracellular N_2_O. Therefore, N_2_O data was not included in bulk or intracellular measurements.

### Respirometry-based biokinetic monitoring

The 'potential' maximum biokinetic rates of NH_3 _oxidation were determined using a short-term (lasting approximately 30 min) batch respirometric assay [32]. The term 'potential' describes non-limiting NH_3 _(initial concentration of 50 mg-N/L) and oxygen concentrations (supersaturated initial concentration of approximately 40 mg O_2_/L, shown previously to be non-inhibitory to NH_3 _oxidation [33]). Maximum NH_3 _oxidation activity per cell was expressed as the specific oxygen uptake rate, sOUR and was calculated by dividing the slope of the respirograms (DO *vs *time) by the cell concentration.

### RNA extraction and purification

40 ml cell suspensions were collected and immediately centrifuged at 4°C and 5000*g for 10 min. The resulting cell-pellets were resuspended and lysed in 1 mL TRIzol^® ^solution (Invitrogen, Carlsbad, CA). RNA was isolated from lysed cell pellets using the TRIzol^® ^RNA isolation protocol (Invitrogen). Subsequent DNA removal and reverse transcription was performed using the QuantiTect^® ^Reverse Transcriptase kit (Qiagen, Valencia, CA).

### Functional gene transcription

Transcript abundance of *amoA*, *hao*, *nirK *and *norB *was quantified by real-time reverse-transcriptase polymerase chain reaction (q-RT-PCR) using previously documented and newly designed primer sets (Table 1). Additional primers for conventional end-point PCR were also designed for *hao, nir*K and *nor*B and used for preparing standard curves for q-RT-PCR (Table 1). Transcription of functional genes was normalized to 16S rRNA concentrations quantified using primers EUBF and EUBR [34]. q-RT-PCR and endpoint PCR were performed in duplicate on an iCycler iQ™5 (Bio-Rad Laboratories, Hercules, CA). A no-template-control was included for each set of PCR and q-RT-PCR reactions. Standard curves for q-RT-PCR consisted of six decimal dilutions of the respective plasmid DNA (corresponding to the four functional genes), containing a given endpoint PCR product. Plasmid concentrations were quantified (Cary 50 UV-Vis spectrophotometer, Varian, Palo Alto, CA) and translated to copy number assuming 660 Da per base pair of double-stranded DNA [35]. Transcript abundance was determined from samples obtained during exponential phase. For exponential phase cultures, sampling time points were 70 hr, 45 hr, and 52 hr for DO concentrations of 0.5, 1.5 and 3 mg/L, respectively, and corresponded to similar cell densities (Figure 3, A4-C4)). For stationary phase cultures, the sampling time points were 165 hr, 116 hr, and 119 hr for DO concentrations of 0.5, 1.5 and 3 mg/L, respectively (Figure 3, A4-C4)). The sampling time points for exponential and stationary phase cultures, which were grown with addded 280 mg NO_2_^-^-N/L were 95 hr, and 143 hr, respectively (Figure 4, D4).

## Authors' contributions

RY performed the experiments and drafted the manuscript. KC conceived of and developed the study, helped to analyze and interpret the results and draft the manuscript. Both authors have read and approved the final manuscript.
